# Analysis of Neutrophil/Lymphocyte ratio and Thiol/Disulfide homeostasis parameters in patients admitted to the emergency department with ischemic stroke

**DOI:** 10.12669/pjms.346.16242

**Published:** 2018

**Authors:** Aysel Begum Yilmaz, Servan Gokhan, Alp Sener, Ozcan Erel

**Affiliations:** 1 *Aysel Begum Yilmaz, MD. Ankara Training and Research Hospital, Department of Emergency Medicine, Ankara, Turkey*; 2 *Servan Gokhan, MD. Yildirım Beyazit University Medical School, Department of Emergency Medicine, Ankara, Turkey*; 3 *Alp Sener, MD. Ankara Ataturk Training and Research Hospital, Department of Emergency Medicine, Ankara, Turkey*; 4 *Ozcan Erel, MD. Yildirim Beyazit University Medical School, Department of Emergency Medicine, Ankara, Turkey*

**Keywords:** Ischemia Modified Albumin, Neutrophil/Lymphocyte Ratio, Stroke, Thiol/Disulfide Homeostasis

## Abstract

**Objectives:**

It is known that the neutrophil/lymphocyte ratio (NLR) is associated with adverse outcomes in ischemic stroke patients. We aimed to reveal the association of NLR and thiol/disulfide homeostasis (TDH) with ischemic stroke patients.

**Methods:**

This study was conducted prospectively at tertiary hospital in emergency department between March 18, 2017 and November 30, 2017. It included 143 patients who were diagnosed with stroke, exhibited no hemorrhage on the Computed Tomography (CT) of the head were included in the study. Complete blood count, biochemical, TDH parameters and Ischemia Modified Albumin (IMA) were studied.

**Results:**

Neutrophil count and NLR were significantly higher in the patient group (p<0.001, p=0.001, respectively). The mean Native Thiol (NT) value of the patient group was 359.9 ± 84.59 μmol/L. The mean Total Thiol (TT) value in the patient group was 399.38 ± 86.06 μmol/L. The NT and TT values in the patient group were significantly lower (NT/TT: p = 0.002/p = 0.007), whereas NLR and IMA were significantly higher in the patient group (p = 0.001/p < 0.001).

**Conclusions:**

Physicians should focus on patients with increased NLR, as these patients appear to represent a population at risk for increased morbidity. We have quantitatively demonstrated in tissue oxidative stress level with TDH parameters. Investigation of these new parameters should be continued for the determination of prognostic significance.

## INTRODUCTION

Cerebrovascular diseases represent the leading causes of both morbidity and mortality in developed countries and despite recent advances in treatment, 20–30% of acute strokes result in mortality, while 70–80% result in permanent sequelae.^1^ These statistics highlight the importance of improving the diagnostic procedures available for acute stroke cases.

Among the numerous leukocyte series, the first cells to infiltrate ischemic brain tissue are the neutrophils. Infiltration begins sometime between the 30th post-ischemic minute and a few hours after the onset of ischemia, and it peaks between the first and third days. It then decreases over time. The infiltration level remains high in ischemic brain tissue for between seven and 15 days.^2^ The number of neutrophils and leukocytes that infiltrate the brain tissue during this period has been shown to be proportional to the severity of the experienced ischemic stroke.^3^ The neutrophil/lymphocyte ratio (NLR) has previously been reported in the literature as a marker of systemic inflammation that can be used to recognize existing subclinical inflammation.^4^ Due to the accessibility and simplicity of the NLR, the test can be rapidly performed on patients admitted to the emergency department who are diagnosed with cerebrovascular disease. Importantly, it can provide an indication of the individual patient’s risk of mortality and morbidity. In the literature, recent studies have demonstrated the relationship between the NLR and ischemic stroke, with the data indicating that the NLR is markedly increased in patients with ischemic stroke, which can be used to predict the patient’s prognosis.^5^,^6^

A physiological equilibrium exists between the oxidant and antioxidant mechanisms, and the disruption of this equilibrium is defined as oxidative stress.^7^ Among the reperfusion damage that is observed following ischemia, an increase in the oxidative end products has previously been found.^8^ Compounds containing the thiol group play a defensive role against oxidative stress due to their reductive characteristics. Following the oxidation of the thiol groups, disulfide bonds are formed through a reversible reaction. Hence, the disulfide bonds can be reduced again into thiol groups. Thus, dynamic thiol-disulfide homeostasis is achieved.^9^ Despite the inflammatory process observed following a stroke and the antioxidant process that develops in response to the consequential ischemic neuronal damage, very little is currently known about the process of the antioxidants, although increasing evidence of abnormal TDH following disease has been found.^10^,^11^ Therefore, it is important to investigate whether there exists a relationship between mortality and morbidity and the thiol levels seen in ischemic stroke, which remains a significant cause of death and disability worldwide.

The present study aimed to determine the diagnostic and prognostic values of both the NLR and TDH in ischemic cerebrovascular disease patients.

## METHODS

This observational study was conducted prospectively with 143 patients who were admitted to the Emergency Medicine Clinic of Ankara Yildirim Beyazit Ataturk Research and Training Hospital between March 18, 2017 and November 30, 2017 and who were subsequently diagnosed with ischemic stroke. The study protocol was approved by the Yildirim Beyazit University Ethics Committee (Dated 14.06.2017 with decision no: 16379996/125). Only those patients who were over 18 years old, diagnosed with stroke based on neurological symptoms at the time of presentation, exhibited no hemorrhage on the Computed Tomography (CT) of the head, and gave consent to participate were included in the study. Some 85 healthy volunteers were also included in the study as the control group. Those patients who did not consent to participate, who were pregnant, who had a body temperature higher than 37.5°C, who were suspected of having an infection, who had a previous history of cerebrovascular disease, who had hemorrhage in CT scan of the head and who had inconsistent information were excluded. The patients’ demographic characteristics at the time of presentation and the findings of the physical examinations were recorded. A complete blood count was performed, and the patients’ biochemical parameters were studied. Using the new method developed by Erel and Neselioglu, the patients’ TDH parameters (Native Thiol [NT], Disulfide [D], and Total Thiol [TT]) were also studied. More specifically, this involved the examination of their disulfide/native thiol (index 1), disulfide/total thiol (index 2), and native thiol/total thiol (index 3) indices. Further, the patients’ ischemia-modified albumin (IMA) and ferroxidase levels were studied. All measurements were performed using an Autocobas 501 auto-analyzer (Roche-Hitachi, Mannheim, Germany).

In summary, the age, neutrophil, lymphocyte, platelet, urea, creatinine, albumin, native thiol, total thiol, disulfide, index 1, index 2, and index 3 values of the patients were recorded. For each patient, the neutrophil to lymphocyte ratio as well as the platelet to lymphocyte ratio were determined, and the NLR and PLR values were calculated. Finally, based on the 28-day mortality data, the patients were divided into two groups, namely the “surviving” and “exitus” groups.

### Statistical Analyses

All the statistical analyses in this study were performed using the SPSS Statistics 16.0 program for Windows. In terms of the evaluation of the data, frequency distributions were provided for the categorical variables, while descriptive statistics were provided for the continuous variables.

For the continuous variables in the study, the Kolmogorov-Smirnov test for normality was used. The Mann-Whitney U test, which is a nonparametric test, was used for the median comparisons among the independent groups, since the values did not meet the normality assumption (p < 0.05). A comparison of the mean of the normally distributed data was performed using the independent samples t-test. For the evaluation of the independent frequency data, the chi-square test was used on 2x2 compartment tables. A receiver operating characteristic (ROC) analysis was performed on the parameters that were significant for mortality, and the area under the curve was calculated. p<0.05 was considered to be statistically significant.

## RESULTS

A total of 228 subjects, comprising 143 patients and 85 healthy volunteers, were included in the study. Gender and age analysis is provided in Table-I. In terms of distribution of gender and age, no significant difference was detected between the two groups (chi-square, p = 0.840; Mann–Whitney U test, p = 0.357).

**Table-I:** Distribution of gender and age in patient and control groups.

			Control group		Patient group		Total
Gender	Female	n	41		67		108
		Ratio	48.2%		46.9%		47.4%
	Male	n	44		76		120
		Ratio	51.8%		53.1%		52.6%
	Total	n	85		143		228
		Ratio	100.0%		100.0%		100.0%

Chi-square: p = 0.840							

Age	Mean	n	Standard deviation	Median		Minimum	Maximum

Control group	69.88	85	12.727	73.00		31	93
Patient group	71.99	143	11.573	73.00		41	98
Total	71.21	228	12.032	73.00		31	98

Mann–Whitney U: p = 0.357 n: Number

Neutrophil count and NLR were significantly higher in the patient group than in the control group (p<0.001, p=0.001, respectively). Complete blood count, TDH parameters and albumin parameters for patient and control groups are shown in Table-II.

**Table-II:** Distribution of parameters in patient and control groups.

	Control group						Patient group						Total						
	Mean	Standard Deviation	Median	Minimum	Maximum	IQR (95% CI)**	Mean	Standard Deviation	Median	Minimum	Maximum	IQR (95% CI)**	Mean	Standard Deviation	Median	Minimum	Maximum	IQR (95% CI)**	p-value *
Neutrophil/μL	5,063	2,329	4,580	1,820	12,740	2,325	6,388	2,964	5,600	900	19,900	3,800	5,864	2,801	5,110	900	19,900	3,048	< 0.001
Lymphocyte/μL	1,768	697	1,750	470	3,690	855	1,720	744	1,615	400	3900	820	1,738	726	1,700	400	3,900	865	0.419
NLR	3.54	3.01	2.57	1.10	19.30	2.28	4.98	4.38	3.64	0.90	33.17	3.50	4.41	3.96	3.13	0.90	33.17	2.71	0.001
NT μmol/L	396.04	85.57	402.00	139.60	575.00	377.6–414.5	359.69	84.59	354.40	61.60	577.10	345.7–373.7	373.24	86.58	376.30	61.60	577.10	361.9–384.5	0.002
TT μmol/L	431.42	84.65	445.10	187.80	619.70	413.2–449.7	399.38	86.06	394.00	184.80	638.90	385.1–413.6	411.32	86.75	410.25	184.80	638.90	400.0–422.6	0.007
D μmol/L	17.71	7.69	18.20	0.50	35.70	11.15	19.51	14.51	16.95	0.45	115.30	14.87	18.83	12.42	17.35	0.45	115.30	13.13	0.961
Index 1	0.0482	0.0266	0.0482	0.0013	0.1726	0.05	0.0675	0.1582	0.0446	0.0013	1.8718	0.04	0.0603	0.1265	0.0455	0.0013	1.8718	0.05	0.004
Index 2	0.0430	0.0212	0.0440	0.0013	0.1283	0.04	0.0493	0.0422	0.0409	0.0013	0.3946	0.03	0.0470	0.0359	0.0417	0.0013	0.3946	0.04	0.004
Index 3	0.9140	0.4250	0.9120	0.7433	0.9974	0.08	0.9004	0,0881	0.9170	0.2108	1.0946	0.06	0.9055	0.0746	0.9149	0.2108	1.0946	0.08	0.008
IMA	66.84	9.57	69.80	49.40	85.60	16.40	79.35	11.92	75.40	62.60	145.80	12.00	74.68	12.63	73.60	49.40	145.80	10.35	< 0.001
Ferroxidase U/L	552.50	199.56	490.50	254.10	1,137.40	259.60	501.32	163.96	481.50	103.00	1,143.80	182.50	520.40	179.36	483.70	103.00	1,143.80	217.07	0.122
Albumin g/dL	4.18	0.59	4.20	2.19	5.34	0.57	4.09	0.40	4.15	2.79	5.00	0.42	4.12	0.48	4.17	2.19	5.34	0.44	0.093

NLR: Neutrophil/Lymphocyte ratio, NT: Native thiol, TT: Total thiol, D: Disulfide, Index 1: D/NT, Index 2: D/TT, Index 3 : NT/TT, IMA: Ischemia Modified Albumin

* Independent samples-t-test for NT and TT; Mann–Whitney U test for others.

** 95% confidence interval for mean NT and TT; interquartile range (IQR) for others.

The mean NT value of the patient group was 359.9 ± 84.59 μmol/L (min/max: 61.6/577.10 μmol/L) (IQR = 345.71–373.68), and the mean NT value of the control group was 396.04 ± 84.59 μmol/L (min/max: 139.6/575) (IQR = 377.58–414.50). While the mean TT value in the patient group was 399.38 ± 86.06 μmol/L (min/max: 184.8/638.9 μmol/L) (IQR = 385.16–413.61), in the control group, this value was 431.43 ± 84.65 μmol/L, (min/max: 187.8/619.7) (IQR = 413.17–449.69). The NT and TT values in the patient group were significantly lower than in the control group (NT/TT: p = 0.002/p = 0.007), whereas NLR and IMA were significantly higher in the patient group than in the control group (p = 0.001/p < 0.001) (Table-II). No statistically significant difference was found between the patient and control groups in terms of lymphocyte, disulfide, ferroxidase or albumin parameters.

Albumin was significantly lower in the group that contained exitus patients (p = 0.021). There was no statistically significant difference between the surviving and exitus patient groups in terms of parameters other than albumin (Tables-III, IV).

**Table-III:** Distribution of NT and TT values in surviving and exitus patient groups.

	Mortality (28 days)	n	Mean	Standard deviation	Standard error of the mean	p-value
NT μmol/L	Surviving	121	365.234711	81.4053978	7.4004907	0.066
	Exitus	22	329.218182	96.8045386	20.6387970	
TT μmol/L	Surviving	121	403.867769	85.7898908	7.7990810	0.144
	Exitus	22	374.690909	85.2664502	18.1788683	

NT: Native thiol, TT: Total thiol, n: Number *Independent samples-t-test

**Table-IV:** Distribution of parameters in surviving and exitus patient groups.

Mortality (28 days)									

	Surviving				Exitus			

	Mean	Median	Minimum	Maximum	Mean	Median	Minimum	Maximum	p-value
Neutrophil/μL	6,239	5,600	900	14,500	7,160	5,700	3,500	19,900	0.450
Lymphocyte/μL	1,740	1,700	400	3,740	1,611	1,450	600	3,900	0.302
NLR	4.70	3.47	0.90	17.67	6.42	4.13	1.18	33.17	0.289
D μmol/L	19.33	16.95	0.45	67.60	20.48	16.80	0.45	115.30	0.624
Index 1	0.06	0.04	0.001	0.33	0.13	0.05	0.001	1.87	0.609
Index 2	0.05	0.04	0.001	0.70	0.06	0.04	0.001	0.39	0.636
Index 3	0.90	0.92	0.60	1.00	0.89	0.92	0.21	0.99	0.709
IMA g/dL	79.55	75.40	62.60	145.80	78.20	78.45	66.90	89.60	0.900
Ferroxidase U/L	493.52	464.80	103.00	1,143.80	544.19	520.70	286.90	806.44	0.112
Albumin g/dL	4.13	4.19	2.96	5.00	3.87	4.02	2.79	4.53	0.021

NLR: Neutrophil/Lymphocyte ratio, NT: Native thiol, TT: Total thiol, D: Disulfide, Index 1: D/NT, Index 2: D/TT, Index 3 : NT/TT, IMA: Ischemia Modified Albumin *Mann–Whitney U test

**Fig.1:**
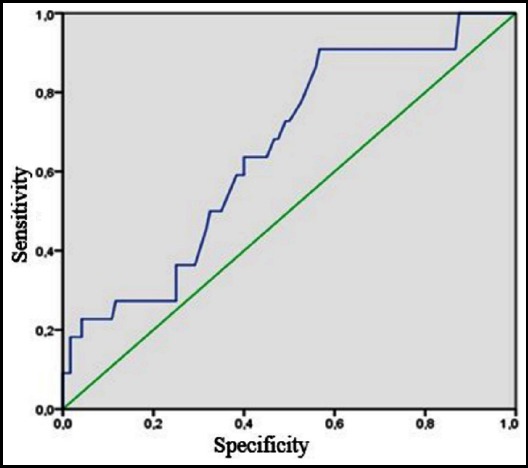
ROC (receiver operating characteristic) analysis: albumin and 28-day mortality.

When Receiver Operator Characteristics curve (ROC) was drawn to evaluate the relationship between albumin and survival at 28 days, the area under the curve was 0.655 (p = 0.021; 95% CI: 0.538−0.772). Based on these results, albumin is not identified as a strong predictive marker for mortality at 28 days (Fig.1).

## DISCUSSION

The role of inflammatory process in the pathogenesis of ischemic stroke is a known and proven mechanism. Neutrophil migration to the brain tissue affected by ischemia has also been shown by histochemical methods. Neutrophil infiltration is considered to play a role in the pathogenesis of ischemic stroke in individuals with increased systemic inflammation. In patients who have a stroke in the presence of infection, the total leukocyte and neutrophil count in circulation and leukocyte-thrombocyte aggregation and activation increases.^3^,^12^ In an empirical study by Del Zoppo et al. it was shown that the volume of infarction was smaller in neutropenic conditions.^13^ Moreover, in patients diagnosed with ischemic stroke, high peripheral leukocyte and neutrophil counts have been shown to be correlated with the volume of ischemic infarction.^3^ In a study by Audebert et al., inflammatory markers, such as body temperature, white blood cell count and CRP, were higher in patients with more severe stroke symptoms and larger ischemic area volume. Indeed, higher rates of successful thrombolysis were reported in patients with a weaker inflammatory response.^14^

On the basis of these findings, clinical studies were conducted to identify inflammatory markers, and NLR was studied. In a long-term study by Suh et al., it was found that high NLR can be an independent risk factor for ischemic stroke.^15^ In a study by Xue et al., NLR was shown to be correlated with stroke severity, primary functional outcome and recurrent ischemic stroke in patients with acute ischemic stroke.^16^ Moreover, it was found by Gokhan et al. that neutrophil and lymphocyte counts and NLR were significantly higher in patients with acute ischemic and haemorrhagic stroke than in transient ischemic attack patients.^5^

In our study, the diagnostic and prognostic value of NLR was investigated in patients diagnosed with ischemic stroke, and it was found that both neutrophil count and NLR were significantly higher in the group diagnosed with ischemic stroke than in the control group. However, since there were no differences between the surviving group and the exitus group in terms of neutrophil count or NLR, there was no evidence for using NLR as a mortality marker. NLR is known to be affected by many different diseases; therefore, there were no data for NLR alone to be used as a marker. Small sample size might have caused this, in which case NLR can be used as an aid to other complementary biochemical tests and imaging techniques. More comprehensive studies are required to reveal NLR’s prognostic value.

Although the exact mechanism is not known, events that lead to ischemia are known to cause the formation of free oxygen radicals at the tissue and cellular levels.^13^ The correlation between oxidative stress and brain damage has been shown in many studies. Moreover, the role of oxidative stress was shown not only in stroke but also in many other diseases.^11^,^17^ In the study by Bektaş et al where TDH was evaluated in ischemic stroke and they found that NT, TT values were statistically significantly lower in the stroke group than the control group. In the study, a negative correlation was also detected between NT and infarction volume.^18^ In our study, in the evaluations performed in terms of TDH parameters, NT and TT values were significantly lower in the patient group. Disulfide was found to be higher in the patient group, although this finding was not statistically significant. Index one and Index twowere significantly higher in the patient group, whereas Index three was significantly lower in the patient group. In terms of NT and TT, these results corroborate the findings of the study by Bektaş et al.^18^

Membrane lipids of neuronal cells are very rich in polyunsaturated fatty acids, which are especially sensitive to free oxygen radicals.^19^ IMA is studied as a nonspecific marker of tissue ischemia. In studies by Abboud et al. and Gündüz et al., a correlation between ischemic brain damage and IMA was shown.^20^,^21^ In our study, we analysed IMA in the patient group and in the control group, and we found that IMA levels were higher in the patient group, a finding that corroborates the literature.

In all parameters that were analysed, albumin was found to be associated with mortality. In terms of albumin, despite the lack of significant difference between the patient and control groups, levels were lower in the exitus group than in the surviving group. However, when the ROC was drawn, albumin could not be regarded as a strong marker of mortality. In the literature, there are studies in which hypoalbuminemia was associated with mortality. In their study of 2,986 people over 12 years of follow-up, Xu et al. found that low serum albumin levels were independently correlated with ischemic stroke (especially cardioembolic and cryptogenic ischemic stroke).^22^

### Limitations of this Study

It comorbid diseases, which may have affected the oxidative stress parameters, were not excluded from the study, and post-treatment oxidative parameters were not studied. The small sample size is another limitation.

## CONCLUSION

Neutrophil count and NLR, which demonstrate the inflammatory process in ischemic stroke (a significant cause of mortality and morbidity), were higher in the patient group. We think that these parameters can be used as an early stage marker in the future. In this study, we have quantitatively demonstrated oxidative stress at the tissue level in ischemic stroke using TDH parameters. In the patient groups, it was found that the level of serum thiol, in particular, decreased, but sufficient data on its prognostic value could not be obtained in this study. Further studies are required to reveal the prognostic value of the relatively new parameters in the literature – namely TT, NT, IMA, disulfide and indexes – in ischemic stroke.

### Authors Contribution

**ABY:** Conceived, designed, did manuscript writing and did data collection.

**AS:** Did statistical analysis and editing of manuscript.

**SG & OE:** Did review, final approval of manuscript.
